# Effect of implementing an evidence-based clinical practice intra-hospital transport guideline for hospitalized children: a type III hybrid implementation-effectiveness study

**DOI:** 10.1186/s43058-026-00962-7

**Published:** 2026-06-06

**Authors:** Yang Li, Yuxia Yang, Jing Hu, Wenchao Wang, Yun Jin, Weijie Shen, Yanhong Zhang, Yingfeng Zhou, Ye Cheng, Jos M. Latour, Ying Gu

**Affiliations:** 1 Pediatric Intensive Care Unit, Children’s Hospital of Fudan University, Shanghai, China; 2 Nursing Department, Children’s Hospital of Fudan University, Shanghai, China; 3 Emergency Department, Children’s Hospital of Fudan University, Shanghai, China; 4 School of Nursing, Fudan University, Shanghai, China; 5 School of Nursing, Faculty of Health, University of Plymouth, Plymouth, UK

**Keywords:** Intra-hospital transport, Children, Pediatrics, Intensive care unit, Pediatric emergency medicine, Implementation science, Effectiveness, Pre-post intervention study

## Abstract

**Background:**

Intra-hospital transfers (IHT) of hospitalized children are unavoidable practices often performed with emergency patients and postoperative patients. Standardizing IHT processes to minimize adverse events might improve children’s outcomes. We developed an evidence-based clinical practice IHT guideline for hospitalized children. The aim of this study was to evaluate the implementation process and the effectiveness of the implementation of this guideline on patient outcomes, healthcare professionals’ knowledge and behavior, and hospital organizational context.

**Methods:**

A type III hybrid effectiveness-implementation design was adopted, using a pre-post intervention trial (January-December 2024). Data of patient demographics, transport-related outcomes, and healthcare providers’ knowledge and compliance were collected. We used the RE-AIM framework to assess effectiveness across four dimensions: Reach, Effectiveness, Adoption, and Implementation. Totally, 110 healthcare professionals conducted 213 IHTs of eligible children (109 children in the pre-intervention group and 104 in post-intervention group).

**Results:**

The Reach outcomes demonstrated that participation among hospitalized children (*n* = 312) was suboptimal at 33% (104/312). No differences were observed between the pre- and post-intervention group regarding gender, disease distribution, or pediatric early warning scores. The implementation showed favorable outcomes in the dimensions Effectiveness, Adoption, and Implementation. Healthcare professionals engagement was 95%, with 86% (19/22) of the implementation strategies successfully completed. Healthcare professionals’ knowledge in the pre-intervention group (*n* = 109) improved from median 40 (IQR 28;52) to median 76 (IQR 64;84) in the post-intervention group (*n* = 104; *p* < 0.001). Clinically, the new guideline reduced adverse events (12 vs 4; *p* = 0.047), reduced the median minutes of bedside handover time from 5 (IQR 3;7) to 4 (IQR 3;5; *p* < 0.001), and improved handover information completeness from median score of 5 (IQR 4;6) to 20 (IQR 12;23, *p* < 0.001). The total transport time increased from 14 to 19 minutes in the post-intervention group (*p* < 0.05), while no significant changes were observed in handover interruptions or post-transfer vital sign stability (*p* > 0.05).

**Conclusion:**

The RE-AIM-based evaluation confirmed that the implementation strategies effectively enhanced healthcare professionals’ knowledge and compliance while reducing adverse events and optimizing handover efficiency. However, the limited patient participation rate and increased transport duration highlight areas requiring further refinement to maximize the guideline’s impact.

**Trial registration:**

ClinicalTrials.gov, NCT06512805. Registered 27 June 2024.

**Supplementary Information:**

The online version contains supplementary material available at 10.1186/s43058-026-00962-7.

## Text box 1. Contribution to the literature

• This study demonstrates that a multifaceted, theory-informed implementation strategy effectively improves healthcare professionals’ adherence of intra-hospital transport guideline, providing practical evidence of barrier-strategy-target alignment in real-world clinical settings.

• Using the RE-AIM framework to systematically assess the implementation process of an evidence-based intra-hospital transport guideline added methodological rigor and enabled replication and comparison across studies.

• This implementation study provides empirical evidence of success factors before and after implementing an evidence-based intra-hospital transport guideline in pediatric hospital settings expanding the global understanding of guideline implementation in diverse healthcare environments.

## Background

Intra-hospital transport (IHT) refers to the transfer and handover of hospitalized patients between different clinical areas within the same healthcare institution to ensure appropriate treatment, monitoring, and care [1]. It is one of the most frequent clinical activities performed in hospitals, typically involving both the coordinated transfer of patients from the original unit to the destination unit and the exchange of clinical information between healthcare professionals (HCPs). The demand and frequency of IHT are high, as it is unavoidable for emergency patients, postoperative patients, and those highly dependent on specialized medical equipment, thereby becoming a routine part of hospital practice. Previous studies have reported that 44% of patients required at least one transport outside the ICU, with 45% of them transported two or more times within the ICUs [2]. The most common destination was for CT scans, followed by the operating room, cardiac catheterization, MRI, and other diagnostic departments [3–5]. Therefore, IHT constitutes an essential component of medical and nursing practice [1].

Despite its necessity, IHT poses considerable challenges. Physiological instability may arise from acceleration, deceleration, positional changes, and exposure to new environments, equipment, and noise, all of which act as stressors affecting physiological parameters of the children [6–8]. Additionally, IHT can provoke psychological distress, including anxiety and fear, due to unfamiliar surroundings, personnel, and equipment [9]. Inadequate transport practices—such as equipment malfunction, insufficient fixation, or monitoring errors—further compromise patient safety [10]. Evidence confirms that adverse events during pediatric IHT are common. A systematic review of IHT of critically ill children reported incidence rates ranging from 0.1% to 75%, with hypoxemia, hypotension, cardiopulmonary resuscitation, accidental catheter dislodgement, and interruption of vasoactive drug infusion being the most frequently observed complications [11]. These risks highlight the importance of standardizing transport procedures to minimize patient harm and improve safety outcomes.

Several guidelines on pediatric transport, both intra- and inter-hospital, have been published internationally, such as the Guidelines for Air and Ground Transport of Critically Ill Children in the United States [12] and pediatric transport protocols in the United Kingdom [13, 14]. In China, the Neonatal Transport Guideline was issued in 2013 [15]. However, these documents primarily focus on inter-hospital transport, offering limited guidance for HCPs engaged in pediatric IHT. To address this gap, our research team developed the clinical practice guideline for intra-hospital transport of hospitalized children (2023 edition) [16], which provides comprehensive recommendations on risk assessment, preparation, equipment and medications, role delineation in different scenarios, standardized procedures, and additional considerations for neonates and children with special conditions.

Although evidence-based implementation studies on IHT remain limited, existing research has primarily focused on adult critically ill patients in emergency settings. These studies have demonstrated that evidence-based interventions, such as structured transport protocols with defined team roles, pre-transport risk assessment, standardized preparation of equipment and medications, and structured bedside handover, can standardize transport practices for HCPs, improve their knowledge, and reduce the incidence of adverse events [17, 18]. Building on this gap, the present study focuses on pediatric IHT to identify current clinical challenges and to evaluate the implementation of the ‘clinical practice guideline for intra-hospital transport of hospitalized children’ (2023 edition) [16]. This study adopts a hybrid type III effectiveness-implementation trial design to assess both implementation outcomes and clinical effectiveness outcomes [19]. Therefore, the aim of this study was to evaluate the impact of the implementation strategies on hospitalized children, HCPs, and clinical environments to improve current clinical practice, standardize pediatric IHT processes, and reduce the incidence of adverse events.

## Methods

### Design and framework

This study employed a type III hybrid effectiveness–implementation design, in which the primary focus is on evaluating implementation strategies while also collecting data on intervention effectiveness [19]. The RE-AIM framework was applied to structure the evaluation process [20]. The RE-AIM framework incorporates both process and outcome indicators in five dimensions; Reach, Effectiveness, Adoption, Implementation, and Maintenance. The dimensions Reach, Adoption, and Implementation were used to evaluate the implementation process of the guideline, while the Effectiveness dimension was used to assess clinical outcomes. Given the limited study duration, the dimension Maintenance (long-term sustainability) and its corresponding indicators were not included.

A quasi-experimental pre–post intervention study design was used to evaluate the impact of implementation strategies on evidence-based practices among HCPs and on the clinical environment. At the same time, the study assessed the effect of guideline implementation on reducing the incidence of adverse events during pediatric IHT and improving other clinical outcomes. The project was conducted over a 12-month period:

- Phase 1: Select evidence (1 month; January 2024)
- Phase 2: Field study (3 months; February- April 2024)
- Phase 3: Pre-intervention data collection (2 months; May-June 2024)
- Phase 4: Implementation of the evidence-based guideline of IHT (4 months; July-October 2024)
- Phase 5: Post-intervention data collection (2 months; November-December 2024)

We followed the reporting guideline “Standards for reporting implementation studies (StaRI)” to report our study [21] (Electronic Supplement Material 1). The study has been registered at the U.S. National Institutes of Health ClinicalTrials.gov (Registration No. NCT06512805).

### Setting

The study was conducted in the Emergency Department (ED) and the Pediatric Intensive Care Unit (PICU) of the Children’s Hospital of Fudan University. The pediatric ED receives approximately 700–800 patient visits per day with around 150 IHT events per month. The PICU has 55 beds, with 120–150 IHT events occurring monthly. Both the ED and PICU are high-frequency sites for IHT and are prone to high-risk incidents. Previous adverse events in these settings, routinely collected through the hospital’s adverse event reporting system, have included patient falls during transport and omission of medications, highlighting the urgent need for standardized pediatric IHT procedures.

### Participants

Participants were categorized into guideline users and target patients. All HCPs who had worked at the study sites for at least one year and were involved in IHT during the study period were considered guideline users. This included transport physicians, transport nurses, ward nurses, attending physicians, and nursing managers. Exclusion criteria were lack of professional qualification or refusal to participate. The target patient population of our study included non-neonatal children transported from the ED to the PICU or from the PICU to general pediatric wards during the study period. Exclusion criteria were children with extracorporeal membrane oxygenation (ECMO) therapy or children with infectious diseases because the evidence-based guideline did not include specific guidance on these criteria.

Based on a preliminary survey conducted by the research team, adherence to evidence-based IHT practices among HCPs in our study setting was 79% [22]. The implementation of the guideline is expected to increase adherence to 95%. Sample size was calculated using the following formula:

$$\begin{aligned}&n = {[{Z_\alpha }\sqrt 2\,\,\,\overline p {\text{ }}(1 - \overline p ) + {Z_\beta }\sqrt p 1(1 - p1)} \cr & + {p2(1 - p2)]^2}/(p1 - p2){{\kern 1pt} ^2}\end{aligned}$$

where *n* represents the sample size per group, Z_α_ = 1.96, Z_β_ = 0.842; p1 = 79%, p2 = 95%，and 1-p1 = 21%, 1-p2 = 5%, *øverline p* =(p1+p2)/2 = 0.87; The calculation yielded *n*≈69. Considering an anticipated 10% loss to follow-up, the final sample size was set at n1 = n2≈76, requiring minimal 76 IHT events for both the pre- and post-intervention phases, totaling a minimal of 152 events.

### Intervention

The clinical practice guideline for intra-hospital transport of hospitalized children (2023 edition) includes 12 recommendations [16] (Electronic Supplement Material 2). The recommendations related to neonates (recommendations 6–8) and special cases (recommendations 9–12) were excluded, and recommendations 1–5 were adopted as the evidence base for this project. In addition, transport experts from the hospital were invited to assess the usability of recommendations 1–5 using the FAME criteria (Feasibility, Appropriateness, Meaningfulness, and Effectiveness) [23]. Experts (*n* = 15) involved in the preparation of the study reached consensus to include all five recommendations as the project’s evidence base.

**Fig. 1:**
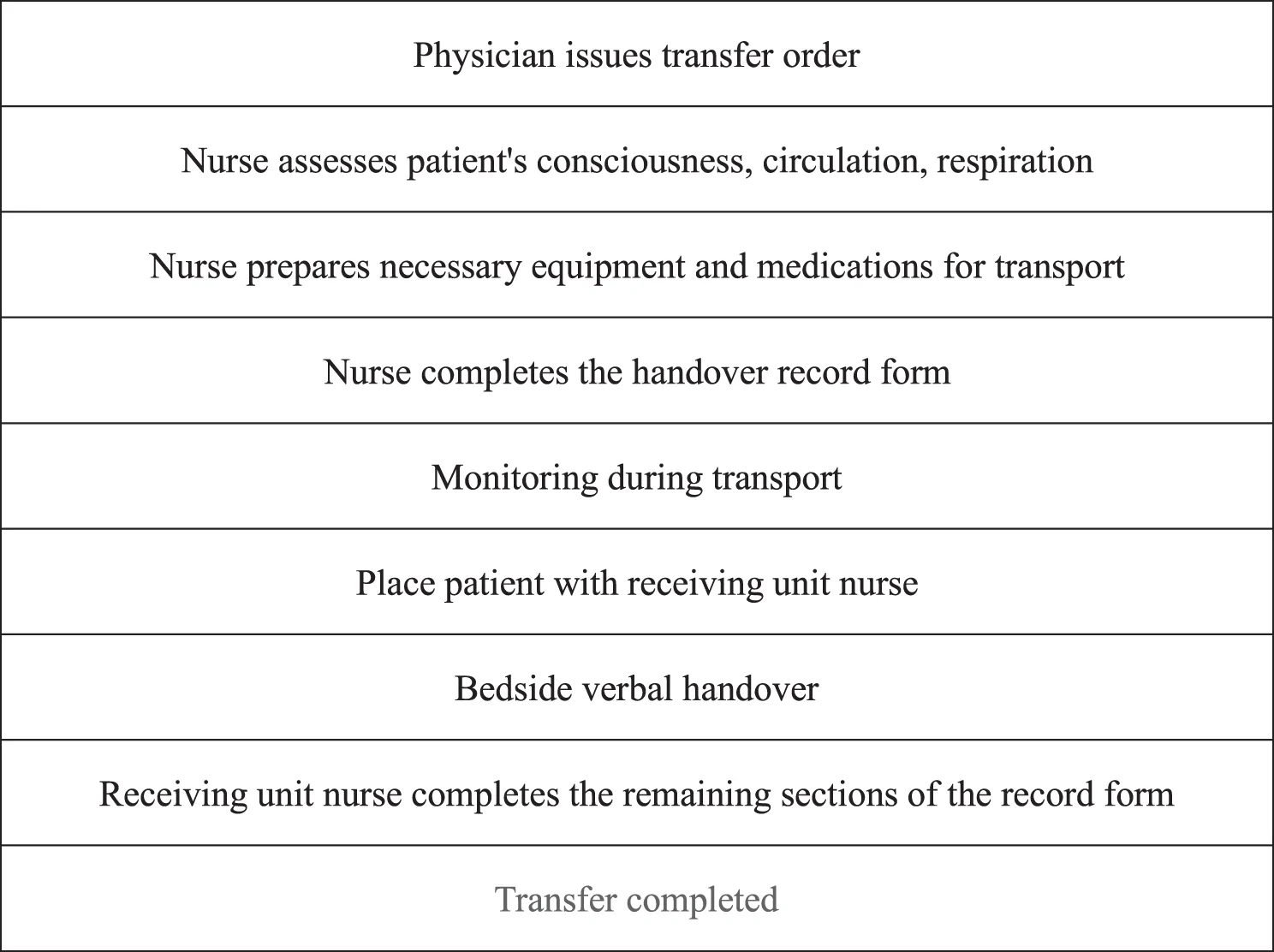
Standard intra-hospital transport workflow

Prior to guideline implementation, HCPs conducted pediatric IHT under routine management and workflow practices in the ED and PICU. The standard transport process is illustrated in Fig. 1.

After guideline implementation, HCPs conducted pediatric IHT in accordance with recommendations 1–5, as detailed in the guideline [16].

### Implementation approach

During the preparation phase, guided by the Consolidate Framework for Implementation Research (CFIR) framework, field observations were conducted over a 3-month period in the ED and PICU using field notes to document current transport practices. In addition, two focus group interviews were conducted with HCPs to explore perceived barriers. These approaches resulted in the identification of 11 barriers to guideline implementation [24]. These barriers included, for example, lack of knowledge and low motivation for change, which informed the selection of tailored implementation strategies. According to the Grol and Wensing Model of Implementation, strategies are more likely to be effective when specifically adapted to the identified barriers [25].

Using the CFIR-ERIC Strategy Matching Tool [26], 44 potential implementation strategies were initially identified. The implementation team then prioritized strategies based on feasibility and importance, selecting 24 strategies for this project. These included: identifying and preparing facilitators, engaging early adopters, involving leadership, informing local opinion leaders, modeling and simulating change, collecting and sharing local knowledge, developing and using quality monitoring tools, conducting local needs assessments, auditing and providing feedback, preparing educational materials, holding educational meetings, and modifying physical structures and equipment. Subsequently, using the Implementation Strategy Reporting Checklist [27], specific change actions were developed along five dimensions: actor, action, action target, temporality, and dosage. Detailed descriptions of these actions are provided in Electronic Supplement Material 3.

### Assessment tools

*Healthcare professionals’ evidence-based IHT practice checklist:* Six steps were used to develop the checklist based on review indicators [28], including: 1) identification of key elements from high-quality evidence; 2) development of indicators based on these elements; 3) prioritization of indicators as “essential” or “recommended”; 4) drafting of the checklist; 5) specification of data collection methods for each indicator; and 6) external expert review. The recommendations 1–5 of the guideline were translated into 15 clear, specific, and measurable items. Adherence to evidence-based practice was calculated as the number of “Yes” responses divided by the total number of “Yes” and “No” responses.

*Healthcare professionals’ IHT knowledge questionnaire:* A self-designed questionnaire consisting of 25 multiple-choice questions covering six domains: definition of IHT (2 questions), communication and explanation (3 questions), risk assessment (7 questions), pre-transport preparation (7 questions), monitoring during transport (2 questions), and post-transport handover (4 questions). Each question was scored 0–4 points, with a total possible score ranging from 0 to 100. The questionnaire was evaluated for content validity by two critical care experts and four nursing experts, resulting in S-CVI/UA = 0.96 and S-CVI/Ave = 0.987.

*Pediatric IHT data collection form:* This form includes patient information (name, hospital ID, diagnosis, date, age, sex, weight, transport risk level) and transport-related information (departing unit, receiving unit, transport team members, departure time from the sending unit, handover start time, arrival time at the receiving unit, adverse events during transport, interventions performed during transport, and staff involved in handover). The form was completed by trained researchers (YL, WJS, YHZ); patient information was obtained from the electronic medical record system, while transport-related data were recorded in real time during transport. Adverse events were defined as clinical deterioration in respiratory, circulatory, or neurological status, as well as equipment-related or process-related issues, and were recorded using a pre-formulated list.

### Outcomes

Based on the RE-AIM framework [20], the implementation process and outcomes of the guideline were evaluated across four dimensions: Reach, Effectiveness, Adoption, and Implementation. The specific evaluation indicators are presented in Table 1.

**Table 1:** Evaluation indicators based on the RE-AIM framework

Indicator	Calculation Formula	Data Collection Time	Data Collection Tool
**1. Reach Dimension: Proportion and representativeness of children receiving the intervention**			
1.1 Patient Project Participation Rate	Numerator: Number of children receiving the intervention; Denominator: Number of children meeting inclusion/exclusion criteria in the post-intervention period.	Post-Implementation	Recruitment data & Patient medical records
1.2 Demographic Representativeness of Participating Patients	Comparison of children receiving evidence-based IHT with all children in the post-intervention period.	Post-Implementation	Recruitment data & Patient medical records
**2. Effectiveness Dimension: Impact of the project on important outcomes**			
2.1 IHT time	Time from transport team departure from sending unit to departure from receiving unit	Recorded during transport	Inpatient IHT data collection form
2.2 Bedside handover time	Time from start of bedside handover to transport team departure from receiving unit	Recorded after arrival at receiving unit	Inpatient IHT data collection form
2.3 Completeness of information handover	Number of items completed on the handover checklist	Recorded after arrival at receiving unit	IHT bedside handover checklist
2.4 Incidence of IHT adverse events	Number of transports with adverse events/Total number of transports during study period	Recorded during transport	IHT data collection form
2.5 Bedside handover interruption rate	Number of interrupted handovers/Total number of bedside handovers	Recorded during transport	IHT data collection form
2.6 Vital signs change	Difference in vital signs before and after transport	Record pre-transport value and first value measured within 15 min of arrival	IHT data collection form
2.7 HCP’s knowledge level of IHT	Knowledge questionnaire score	Tested pre- and post-intervention	HCP IHT knowledge questionnaire
**3. Adoption Dimension: Proportion and representativeness of settings and practitioners willing to initiate the program**			
3.1 Proportion of units participating in the project	Number of units actually participating/number of units eligible for recruitment	Throughout project	Implementation plan schedule
3.2 Proportion of HCPs engaged in the project	Number of HCPs actually participating/number of HCPs eligible for recruitment	Throughout project	Implementation plan schedule
**4. Implementation Dimension: Degree to which the program is implemented as intended and the associated costs**			
4.1 HCPs adherence to EBP behaviors	Number of times the indicators are correctly executed/total number of times the indicator is reviewed × 100%	Pre- and post-implementation	HCP IHT evidence-based practice behavior audit form (15 items)
4.2 Completion rate of implementation strategies as planned	Number of times actually executed/number of times planned	During intervention phase	Implementation plan schedule

EBP = Evidence-based practice; HCP = Healthcare Professionals; IHT = Intra-hospital transport

### Analysis

All statistical analyses were performed using SPSS version 25.0. Continuous variables with a normal distribution were summarized as mean ± standard deviation (SD). The categorical variables were presented as frequencies and percentages. Group comparisons were conducted using t-tests, chi-square tests, or Fisher’s exact tests, as appropriate. Continuous variables with a non-normal distribution were described using median and interquartile range (IQR) and compared using the Wilcoxon rank-sum test.

## Results

### Participants

During the pre- and post-intervention period, there were 110 HCPs performing IHTs. After the pre-intervention period, four nurses were transferred (three nurses had position changes and one nurse resigned), resulting in 106 HCPs performing IHTs in the post-intervention group. Apart from changes in the attending physicians, responsible for transporting pediatric patients from the ED to the PICU, all other staff remained at their same unit. No differences were observed between the two groups in terms of baseline characteristics (Table 2).

**Table 2:** Characteristic of healthcare professionals

	Pre-intervention Group (n = 110) n (%)	Post-Intervention Group (n = 106) n (%)	t/χ^2^	p
Gender	97 (88.2)	92 (86.8)	0.095	0.758
Age in years; mean; SD	33.3; 6.8	33.3; 7.1	0.011	0.991
Profession			0.02	0.887
Physician	23 (20.9)	23 (21.7)		
Nurse	87 (79.1)	83 (78.3)		
Years of Experience			0.192	0.979
1～3	23 (20.9)	20 (18.9)		
4～6	25 (22.7)	24 (22.6)		
7～10	20 (18.2)	21 (19.8)		
＞10	42 (38.2)	41 (38.7)		
Professional Title			0.027	0.986
Junior	74 (67.3)	72 (67.9)		
Intermediate	28 (25.5)	26 (24.5)		
Senior	8 (7.3)	8 (7.5)		
Education Level			0.268	0.994
Associate Degree	20 (18.2)	19 (17.9)		
Bachelor’s Degree	67 (60.9)	64 (60.4)		
Master’s Degree	20 (18.2)	19 (17.9)		
Doctoral Degree	3 (2.7)	4 (3.8)		
Department			0.014	0.906
Emergency	17 (15.5)	17 (16.0)		
PICU	93 (84.5)	89 (84.0)		

PICU = Pediatric Intensive Care Unit; SD = Standard Deviation

The 110 HCPs conducted IHTs of 213 eligible pediatric patients. Of these, 109 patients were assigned to the pre-intervention group and 104 to the post-intervention group. No differences were observed between the both groups in terms of sex, disease distribution, PEWS scores, or transport destination. Statistical difference was observed in age of the children (Table 3).

**Table 3:** Characteristics of children

	Pre-intervention Group (n = 109) n (%)	Post-Intervention Group (n = 104) n (%)	χ^2^/Z	p
Gender	47 (43.1)	45(43.3)	0.000	0.982
Age			12.364	0.002
Infant (0–12 months)	17 (15.6)	2 (1.9)		
Child (1-12 years)	74 (67.9)	84 (80.8)		
Adolescent (13–18 years)	18 (16.5)	18 (17.3)		
Transfer Destination			3.563	0.168
Internal Medicine	44(40.4)	31 (29.8)		
Surgery	47 (43.1)	58 (55.8)		
PICU	18 (16.5)	15 (14.4)		
Disease Distribution			13.628	0.136
Circulatory System Diseases	3 (2.8)	1 (1.0)		
Nervous System Diseases	27 (24.8)	38 (36.5)		
Urinary System Diseases	11 (10.1)	3 (2.9)		
Digestive System Diseases	15 (13.8)	13 (12.5)		
Respiratory System Diseases	5 (4.6)	11 (10.6)		
Skeletal System Diseases	5 (4.6)	2 (1.9)		
Oncological Diseases	19 (17.4)	14 (13.5)		
ENT Diseases	5 (4.6)	4 (3.8)		
Critical Illness	18 (16.5)	15 (14.4)		
Other	1 (0.9)	3 (2.9)		
PEWS Score [M(P_25_,P_75_]	0 (0,0)	0 (0,0)	0.539	0.590

ENT = Ear, Nose and Throat; PEWS = Pediatric Early Warning Score; PICU = Pediatric Intensive Care Unit

### Reach

Data of 312 children were retrieved from the ED and PICU records in the two-month post-intervention period. Of these, data of children of 208 IHTs were not recorded due to transport outside office hours or children with exclusion criteria. Totally, 104 hospitalized children received the guideline-based IHT resulting in a participation rate of 33.3% (104/312). Comparisons between children in the post-intervention group and all children who received IHT showed no statistically significant differences in gender, disease distribution, or PEWS scores while age was significantly different (Table 4).

**Table 4:** Characteristics between children in the post-intervention group and all children in the implementation period

	Post-intervention group (n = 104) n (%)	All Eligible Patients (n = 312) n (%)	χ^2^/Z	p
Gender	45 (43.3)	136 (43.6)	0.003	0.954
Age			22.125	<0.001
Infant	2 (1.9)	63 (20.2)		
Child	84 (80.8)	187 (59.9)		
Adolescent	18 (17.3)	62 (19.9)		
Disease Distribution			9.558	0.387
Circulatory System Diseases	1 (1.0)	3 (1.0)		
Nervous System Diseases	38 (36.5)	103 (33.0)		
Urinary System Diseases	3 (2.9)	9 (2.9)		
Digestive System Diseases	13 (12.5)	59 (18.9)		
Respiratory System Diseases	11 (10.6)	17 (5.4)		
Skeletal System Diseases	2 (1.9)	7 (2.2)		
Oncological Diseases	14 (13.5)	26 (8.3)		
ENT Diseases	4 (3.8)	11 (3.5)		
Critical Illness	15 (14.4)	69 (22.1)		
Other	3 (2.9)	8 (2.6)		
PEWS Score: Median (P_25_,P_75_)	0 (0,0)	0 (0,0)	1.325	0.185

### Effectiveness

The Effectiveness dimension included seven outcome indicators. Compared with the pre-intervention group, the post-intervention group, the IHTs showed significantly improved performance in transport duration, completeness of information handover, overall IHT knowledge, and each knowledge subdomain (*p* < 0.001). The handover-time and the incidence of adverse events during IHT were significantly lower in the post-intervention group (Table 5).

**Table 5:** Results of the effectiveness dimension pre- and post-intervention groups

	Pre-intervention group (n = 109)	Post-intervention Group (n = 104)	Z/χ^2^	p
IHT time in minutes (median; IQR)	14 (12,19)	19 (16,23)	4.660	＜0.001
Bedside Handover time in minutes (median; IQR)	5 (3,7)	4 (3,5)	3.756	＜0.001
Completeness of Information Handover (median; IQR)	5 (4,6)	20 (12,23)	11.604	＜0.001
Bedside Handover Interruption n (%)			1.861	0.172
Yes	26 (23.9)	17 (16.3)		
No	83 (76.1)	87 (83.7)		
IHT Adverse Events n (%)			3.930	0.047
Yes	12 (11.0)	4 (3.8)		
No	97 (89.0)	100 (96.2)		
Vital signs change before/after IHT	median (IQR)	median (IQR)		
Temp (°C)	0.2 (0.1,0.4)	0.3 (0.1,0.5)	1.626	0.110
Pulse beats/min	9 (3,19)	8 (3.5,16)	0.345	0.731
RR breaths/min	3 (2,6)	3.5 (2,6)	0.522	0.603
Diastolic BP mmHg	6 (3,11)	7 (3,12.5)	1.096	0.274
Systolic BP mmHg	8(3,13)	8(4,15.5)	0.904	0.367
SpO2%	1 (0,1)	1 (0.5,1)	1.140	0.255
PEWS Score	0 (0,1)	0 (0,1)	0.465	0.643
Knowledge Questionnaire	median (IQR)	median (IQR)		
Definition of Intra-hospital Transport	0 (0,4)	8 (4,8)	7.238	＜0.001
Communication and Explanation Before Transport	8 (4,8)	12 (8,12)	6.804	＜0.001
Risk Assessment Before Transport	12 (8,20)	24 (20,28)	8.405	＜0.001
Transport Personnel, Equipment, Medication Preparation	8 (4,12)	20 (12,24)	8.540	＜0.001
Monitoring During Transport	4 (0,8)	8 (4,8)	6.104	＜0.001
Handover After Transport	8 (4,8)	12 (8,16)	8.429	＜0.001
Total Score	40 (28,52)	76 (64,84)	10.728	＜0.001

BP = Blood Pressure; IHT = Intra-Hospital Transport; IQR = Inter Quartile Range; RR = Respiratory Rate; Temp = Temperature; PEWS = Paediatric Early Warning Score

Before the implementation period, 12 adverse events were recorded. Nine were related to omission of patient belongings and own diary formula, and medications during transport, while three adverse events involved wrong-floor transfers, PICC line occlusion, and vomiting during transport. After the implementation period, four adverse events were recorded, all associated with omitted items by transport HCPs. No differences were observed between groups in terms of bedside handover interruptions or changes in vital signs (Table 5).

### Adoption

During guideline implementation, both the ED and PICU participated in executing the implementation strategies and transporting patients using the evidence-based IHT guideline. Patients from the PICU were transported to 22 general wards of the total 24 wards of our hospital, accounting for 92% of all eligible wards. A total of 106 of the 110 (96%) HCPs from the ED and PICU engaged in the implementation strategies and guideline implementation during IHT.

### Implementation

The HCP behavior audit form included 15 items. The items 1 and 2 had 100% adherence by HCPs in the pre- and post-intervention groups. Items 3, 4, 6–9, and 11 had 0% adherence of HCPs in the pre-intervention group. After implementing the intervention, the HCPs’ adherence rate increased > 80% on the items 3, 6, 8, 9, and 11. The items 4 and 7 reached an adherence rate of <60%. Adherence for items 5 and 10 improved significantly after the implementation of the evidence-based IHT guideline. No differences were observed for items 12–15 in the pre- and post-intervention groups (Table 6).

**Table 6:** Adherence of HCPs based on the IHT evidence-based practice behavior audit form

Audit Indicator	Pre-intervention group (*n* = 109) n (%)	Post-intervention group (*n* = 104) n (%)	χ^2^	P
1	109 (100)	104 (100)		
2	109 (100)	104 (100)		
3	0	102 (98.1)	205.140	＜0.001
4	0	60 (57.7)	87.545	＜0.001
5	90 (82.6)	102 (98.1)	14.402	＜0.001
6	0	99 (95.2)	193.867	＜0.001
7	0	45 (43.3)	59.797	＜0.001
8	0	91 (87.5)	166.515	＜0.001
9	0	92 (88.5)	169.736	＜0.001
10	25 (22.9)	97 (93.3)	107.588	＜0.001
11	0	91 (87.5)	166.515	＜0.001
12	77 (80.6)	80 (76.9)	1.083	0.298
13	101 (92.7)	102 (98.1)	2.384	0.123
14	100 (91.7)	95 (91.3)	0.011	0.917
15	108(99.1)	104 (100)	0	1

Audit Indicators: 1) Physicians inform the child’s family about the risks and benefits of transport, and the decision to proceed with transport is made jointly by physicians and parents; 2) Before transfer, the sending nurse (or physician) communicates transfer details by phone with the receiving nurse (or physician); 3) Before transfer, the sending nurse conducts an intra-hospital transport risk assessment using the institutional risk assessment tool; 4) Nurses inform physicians of the child’s transport risk level and confirm jointly with them; 5) Before transfer, the sending nurse must plan the transport route; 6) Before transfer, the sending nurse prepares for transport according to the transport preparation checklist; 7) Based on the child’s transport risk level, physicians and nurses are allocated according to the standard staffing requirements; 8) Based on the child’s transport risk level, the sending nurse prepares the required transport equipment according to the equipment checklist; 9) Based on the child’s transport risk level, the sending nurse prepares the required transport medications according to the medication checklist; 10) Before bedside handover begins, participating nurses should suspend other tasks and focus on handover; 11) During bedside handover, participating nurses should use the intra-hospital transfer handover checklist; 12) After bedside handover, participating nurses should avoid interrupting the handover process; 13) At the end of handover, the sending physician or nurse should ask whether the receiving staff have any questions and allow sufficient time for clarification; 14) After confirming together that the handover information is correct, the transferring and receiving nurses may conclude the handover; 15)The transferring and receiving nurses should clearly document the child’s condition and medical interventions during transport in the medical record

During the preparation phase, 22 implementation strategies were developed based on 11 identified barriers (excluding 2 guiding strategies). Of these, 19 strategies were successfully implemented. Three strategies (nr 2, 3, 21) were not completed as planned, resulting in an overall completion rate of 86% (19/22) (Table 7 and Electronic Supplement Material 3).

**Table 7:** Implementation strategy Completion status

Implementation Strategy	Time	Planned Frequency	Completion Status
1. Conduct local needs assessment	2024.7.1–7.31	Continuous	Analyzed 1 previously reported intra-hospital transport risk event; Conducted participatory observation of current transport practices; Collected 14 transport-related suggestions via questionnaire
2. Conduct local consensus discussions	2024.7.1–10.31	At least 2 times	Completed once
3. Assess for readiness and identify barriers and facilitators	2024.7.1–7.31 2024.10.1–10.6	2 times	Completed once
4. Tailor strategies	2024.8.1–8.18	Continuous	Developed 24 implementation strategies based on identified barriers
5. Change physical structure and equipment	2024.8.1- 2025.6.30	Continuous	Updated ward layout map, optimized PICU-2 transport route; widened 1 doorway, repaired 1 steep slope; added 5 pulse oximeters
6. Develop a formal implementation blueprint	2024.8.12–10.31	Weekly	Developed 1 scheduling plan; 10 group progress reports completed
7. Develop and implement tools for quality monitoring	2024.8.12–10.31	Continuous	Developed audit checklist and adverse event reporting form, incorporated into Nursing Department quality control system
8.Involve executive boards	2024.8.1- 2025.6.30	Continuous	Nursing Department, Medical Affairs Department, and department directors involved in this change project
9. Develop educational materials	2024.8.19–8.31	Continuous	Developed 4 reference tools for intra-hospital transport, 3 e-learning materials, updated 1 policy document, and 2 scenario-based simulation cases
10. Identify and prepare champions	2024.8.26- 2025.6.30	Continuous	Identified 19 facilitators, completed theoretical training, clarified responsibilities
11. Identify early adopters	2024.8.26–10.31	Continuous	Identified 12 early adopters
12. Inform local opinion leaders	2024.8.26- 2025.06.30	Continuous	Identified 28 opinion leaders, completed theoretical training, clarified responsibilities
13. Make training dynamic	2024.8.26–10.31	Continuous	Distributed educational materials, conducted theoretical training and on-site drills
14. Distribute educational materials	2024.8.26–9.30	Continuous	Distributed educational materials to ED, ICUs, and 28 general wards
15. Conduct educational meetings	2024.8.26–9.30	Weekly	Completed 13 in-person theoretical training sessions; 1 hospital-wide lecture
16. Conduct educational outreach visits	2024.9.1–10.31	Continuous	Received guidance and training from 1 simulation training expert; facilitators, opinion leaders, and early adopters provided clinical guidance
17. Capture and share local knowledge	2024.9.1–10.31	Weekly	Completed 7 participatory observations and 1 case-sharing session during intervention period
18. Audit and provide feedback	2024.9.1–10.31	Weekly	Completed 7 audits during intervention period
19. Facilitate relay of clinical data to providers	2024.9.1- 2025.6.30	Continuous	Completed 2 data feedback sessions, including posting on-site information and sharing via work group chats
20. Purposefully re-examine the implementation	2024.9.1- 2025.6.30	Every 3 months	Held 3 additional ward-based educational meetings
21. Model and simulate changes	2024.9.23–10.27	Weekly	Planned simulation drills not yet started
22. Shadow other experts	2024.9.23–10.31	Continuous	Facilitators and early adopters conducted 18 model transport demonstrations

## Discussion

This study used a type III hybrid effectiveness–implementation design and constructed a multidimensional evaluation system based on the RE-AIM framework, with a primary focus on assessing the effects of implementation strategies and a secondary focus on clinical outcomes associated with guideline implementation. The results showed that the majority of the HCPs participated in the implementation of the evidence-based guideline of IHT. We also observed that most of the implementation strategies were completed. The implementation of the strategies significantly improved healthcare professionals’ knowledge and adherence to evidence-based practices. We also observed a reduction in the incidence of adverse events during IHT, bedside handover time, and enhancing the completeness of information handovers.

The dimension Reach of the RE-AIM framework reflects how accessible and representative an intervention is among the target population [20]. In our study, only 33% of eligible pediatric patients participated, mainly due to limited daytime data collection. Importantly, as the guideline was implemented uniformly among healthcare providers, patients outside the audited periods were likely to have also received standardized transport care, although their data were not captured. This may have introduced potential selection bias and could affect the representativeness of the findings. However, enrolled patients were comparable to the target population in sex, disease distribution, and PEWS scores, indicating a reasonable level of representativeness. Evidence suggests that greater alignment between enrolled and target populations facilitates broader guideline adoption [20]. Therefore, future staged scale-up studies should include more diverse patient populations to enhance generalizability and further validate the effectiveness of the intervention. The engagement of the HCPs was high as measured in the dimension Adoption. This likely resulted from the support of the leadership of the wards and hospital. Leadership provides guidance, coordination, and motivation, which are essential for change [29]. Engaging facilitators, early adopters, and opinion leaders, combined with educational meetings and materials, enhanced staff engagement and guideline uptake [30–32]. Overall, our findings highlight that evaluating both Reach and Adoption is critical; effectiveness alone does not ensure broad implementation, and attention to implementation processes is necessary to extend the intervention’s impact.

Evidence-based guidelines for IHT offer a structured framework for optimizing safety and resource use. Prior implementation studies and quality improvement projects have shown that guideline implementation improved overall transport quality, reduced adverse events, and enhanced the completeness of information transfer without prolonging handover time [33–35]. Our findings align with these results but also reveal notable differences. In particular, guideline-based processes were associated with a longer overall transport duration. This extension was primarily due to three additional steps: pre-transport risk assessment, systematic preparation of personnel, equipment, and medications, and the use of SBAR checklists during handover. Although these steps lengthened the process, they reduced omissions, minimized risks from deliveries, and improved handover efficiency by ensuring that critical information was prepared in advance. This reflects a trade-off between improved patient safety and increased time expenditure. Clinically, the reduction in adverse events may justify the additional time, whereas operationally, longer transport duration may affect workflow efficiency and resource use. Future studies should explore strategies to balance safety and efficiency, and assess whether transport time decreases as healthcare professionals gain familiarity with standardized procedures.

Implementation science research indicates that multilevel strategies are generally more effective than a single strategy, as they address multiple barriers in clinical practice and enhance implementation outcomes [36]. In our study, key elements of evidence translation included assessing transport risk levels, allocating personnel/equipment/medications according to risk, and conducting SBAR-based bedside handovers. Before implementation, HCPs’ knowledge and adherence to evidence-based IHT practices were low. Following the introduction of multilevel strategies—incorporating stakeholder engagement, education, process audits, and data feedback—both knowledge and adherence improved, consistent with prior research in emergency critical care transport [37].

Adherence to our indicator “allocate personnel according to transport risk” remained low in the post-intervention period likely due to structural shortages of qualified PICU staff and conflicts between current departmental roles and transport protocols. In routine practice, transfers from PICU to general wards are generally stable, and nurses primarily manage transport, with physician involvement limited to cases where receiving units require detailed clinical handover. Both our study and previous research indicated that physician non-participation in such low-risk transfers does not increase adverse events [38, 39]. Despite improved adherence through intensive implementation strategies, further rigorous non-inferiority trials are needed to evaluate whether nurse-led IHT for low-risk patients is as safe as physician-accompanied transport for hospitalized children. Such evidence could inform optimization of resource allocation and enhancing efficiency while maintaining patient safety.

Previous studies have shown that multi-level implementation strategies are commonly used, often involving 10–16 strategies when applying the CFIR–ERIC matching tool [40–43]. In our study, a larger number of strategies (*n* = 24) were developed, primarily due to the higher number of identified barriers and the need to tailor strategies to the local context. While such comprehensive multi-level strategies may enhance implementation, they may also increase complexity and burden, potentially affecting feasibility and execution [20, 24].

In our study, 86% of the implementation strategies were completed, while 3 were not fully executed, mainly due to resource limitations, low perceived value when evidence had already been integrated into routine care, and the burden associated with multiple strategies. These gaps in implementation may have contributed to the suboptimal adherence observed for certain indicators (e.g., items 4 and 7) and may have attenuated the overall effectiveness of the intervention. These findings highlight the importance of maintaining adaptive implementation plans that are guided by stakeholder preferences and local priorities [44]. Moreover, the effectiveness of any intervention depends not only on its intrinsic efficacy but also on the fidelity and validity of the strategies supporting it [45]. Because not all strategies contribute equally to success [46], systematic documentation and monitoring of their use is essential to identify those with the greatest impact. By employing a structured checklist capturing key elements such as actors, actions, and targets, our study provided a transparent account of implementation processes and offered a foundation for future optimization.

Some limitations need to be addressed. Our study was conducted at a single center with a relatively small sample size, which may limit the generalizability of the findings. In addition, the use of a pre–post quasi-experimental design without a control group limits the ability to establish causal relationships between the intervention and the observed outcomes. However, this design was chosen to reflect real-world clinical practice and to facilitate the implementation of the guideline in a complex healthcare setting. Improvement in adherence for some audit indicators was modest, indicating that implementation strategies require further optimization. Additionally, the Maintenance dimension of the RE-AIM framework was not assessed in our study. As a result, the sustainability of the implementation strategies beyond the short observation period remains unknown. We were unable to measure whether improvements in adherence would be sustained at 6 or 12 months after implementation, or whether HCPs’ compliance might decline over time. Future studies should incorporate longer follow-up periods and repeated assessments to evaluate the long-term sustainability of the intervention and its integration into routine clinical practice.

## Conclusions

Overall, the multi-level implementation strategies and our newly developed guideline demonstrated positive effects across the four dimensions of the RE-AIM framework, facilitating improvements in HCPs’ adherence to evidence-based practices and showing an adequate integration with routine transport processes. The application of our evidence-based IHT guideline shortened bedside handover time, improved both the standardization rate and completeness of bedside handover, reduced the occurrence of adverse events during intra-hospital transport, and enhanced the overall quality of pediatric intra-hospital transport. As an innovative application of implementation science in the field of pediatric IHT, our study not only verified the feasibility of translating evidence-based guidelines into clinical practice but also provided methodological insights into promoting evidence implementation in real-world clinical contexts. The findings offer empirical evidence for improving the quality of pediatric IHT and serve as a valuable reference for the broader implementation of pediatric transport guidelines in other healthcare settings. Further research is required to demonstrate the long-term effectiveness of using the evidence-based IHT guideline. In addition, the relatively limited patient participation rate suggests the need to include a more representative patient population in future studies, while the increased transport duration warrants further investigation to evaluate the balance between improved safety and operational efficiency.

## Electronic supplementary material

Below is the link to the electronic supplementary material.

Supplementary material 1

Supplementary material 2

Supplementary material 3

## Data Availability

The data that support the findings of this study are available from the corresponding author, upon reasonable request.
