# Supplementary material for: Effect of implementing an evidence-based clinical practice intra-hospital transport guideline for hospitalized children: a type III hybrid implementation-effectiveness study
Source: Implement Sci Commun. 2026 Jun 6;7:168. doi: 10.1186/s43058-026-00962-7 (PMC13508307; doi:10.1186/s43058-026-00962-7)
Supplement: Supplementary file 2 [file 43058_2026_962_MOESM2_ESM.docx]

**Electronic Supplement Material 2**

**Recommendation 1** Perform risk assessment prior to intrahospital transport of hospitalized children. (1D)

**Suggested tools and methods for applying the recommendation:** Establishing transport risk stratification is a prerequisite for allocating appropriate personnel, equipment, and medications for intrahospital transport. Existing studies [10–20] indicate that researchers have consciously applied structured tools to evaluate the risks associated with pediatric intrahospital transport. However, as these tools were originally developed to assess the risk of clinical deterioration rather than specifically for transport, there is no broad consensus on how the results should be interpreted in relation to transport risk levels, which has become a barrier in evidence translation.

Based on the available evidence, the guideline working group developed a consultation questionnaire on transport risk stratification and related arrangements, which was distributed to 16 EtD experts. Fifteen experts responded, and 14 (87.5%) agreed that intrahospital transport risk should be classified into three levels:

Level I: Critically ill, with unstable respiration and/or circulation, requiring or potentially requiring advanced life support during transport.

Level II: Critically ill, but with relatively stable respiration and circulation, with a lower likelihood of deterioration during transport.

Level III: Clinically stable, with little likelihood of deterioration during transport.

**Recommendation 2** Assign appropriate personnel and prepare necessary equipment and medications according to the transport risk level. (2D)

**Suggested tools and methods for applying the recommendation：**Based on the expert opinions collected through the consultation questionnaire regarding transport arrangements, the guideline working group drafted a checklist of personnel qualifications, equipment, and medications to be allocated according to the three transport risk levels, and differentiated between neonates and children/adolescents. The draft was reviewed item by item by the 15 members of the EtD expert panel, and the checklist was unanimously approved (Table 1).

Table 1. Personnel Qualifications, Equipment, and Medications According to Transport Risk Levels

| **Population** | **Category** | **Level I Risk** | **Level II Risk** | **Level III Risk** |
| --- | --- | --- | --- | --- |
| **Neonates** | **Qualifications** | Licensed physician or nurse, trained in neonatal resuscitation techniques, capable of providing advanced life support; ≥3 years clinical experience, ≥2 years in neonatology. | Licensed physician or nurse, trained in neonatal resuscitation techniques, capable of providing advanced life support; ≥1 year in neonatology. | Licensed physician or nurse, trained in neonatal resuscitation techniques, capable of providing advanced life support; ≥6 months in neonatology. |
|  | **Equipment** | Bag-mask/T-piece resuscitator, portable suction device with catheters, transport incubator with ventilator, oxygen supply ≥10 L, intubation kit, stethoscope, transport monitor, infusion/syringe pumps, IV access supplies, chest drainage kit if needed, gastric tube. | Bag-mask/T-piece resuscitator, portable suction device with catheters, transport incubator, oxygen supply ≥10 L, intubation kit, stethoscope, transport monitor, infusion/syringe pumps, IV access supplies. | Bag-mask/T-piece resuscitator, portable suction device with catheters, transport incubator, oxygen supply ≥4 L, pulse oximeter. |
|  | **Medications** | Epinephrine, 0.9% sodium chloride injection, midazolam, dexamethasone, 10% glucose injection. | Epinephrine, 0.9% sodium chloride injection, 10% glucose injection. | Epinephrine, 0.9% sodium chloride injection. |
| **Children & Adolescents** | **Qualifications** | Licensed physician and nurse, trained in pediatric resuscitation techniques, capable of providing advanced life support; ≥2 years in specialty, ≥6 months in ICU. | Licensed physician and nurse, trained in pediatric resuscitation techniques, capable of providing advanced life support; ≥1 year in specialty. | Licensed physician and nurse, trained in pediatric resuscitation techniques, capable of providing advanced life support; ≥1 year in pediatrics. |
|  | **Equipment** | Transport bed, transport monitor, oxygen supply ≥10 L, infusion/syringe pumps, IV access supplies, bag-mask resuscitator, portable suction device, intubation kit, oropharyngeal airway, transport ventilator if required, restraints if required. | Transport bed, transport monitor, oxygen supply ≥10 L, infusion/syringe pumps, IV access supplies, bag-mask resuscitator, portable suction device. | Pulse oximeter, oxygen supply ≥4 L if needed, bag-mask resuscitator. |
|  | **Medications** | Epinephrine, 0.9% sodium chloride injection, midazolam, dexamethasone. | Epinephrine, 0.9% sodium chloride injection, midazolam. | Epinephrine, 0.9% sodium chloride injection. |

**Recommendation 3** Clinical staff should carry out intrahospital transport of hospitalized children according to role responsibilities in different transport scenarios. (1D)

**Recommended Tools and Methods for Applying Expert Recommendations:** According to the EtD expert consensus (16 experts, 100% agreement), intra-hospital transport may occur in various clinical scenarios, including but not limited to: transport from the emergency department to the ward or ICU, from the operating room to the ICU, from the operating room to the post-anesthesia care unit (PACU), from the PACU to the general ward or ICU, from the general ward to the operating room, from non-ICU areas to the ICU, and from the ICU to the operating room or general ward (Figures 1). It is recommended that transport be conducted according to the intra-hospital transport flowcharts corresponding to different clinical scenarios.

The expert consensus also recommends the use of standardized intra-hospital transport handover checklists to ensure safe and effective patient transfer. These checklists include a transport preparation checklist, an intraoperative telephone communication transport checklist, a postoperative face-to-face handover checklist and instructions, and a non-surgical transport face-to-face handover checklist.

Figures 1 PICU to Target Department Transfer Flowchart

| **Timeline** | **Transfer & Handover Process** | **Key Step** | **Implementation Details** |
| --- | --- | --- | --- |
| **Planning Phase** | Confirm Patient Destination  ↓ |  |  |
|  | Obtain Parental Informed Consent  ↓ |  |  |
| **Before Leaving ICU** | ICU Nurse communicates with Ward Nurse via phone  ↓ | **1st Communication** | ▸ Review patient's basic information & current status.  ▸ Confirm planned departure time. ▸ Confirm ward bed availability.  ▸ Ask if the ward nurse has any questions. |
|  | ICU Doctor & Nurse jointly assess transfer risk  ↓ | **Risk Assessment** | ▸ Assess transfer risk based on the 3-level risk grading system. |
|  | ICU Doctor & Nurse jointly plan the transfer  ↓ | **Route Planning** | ▸ Plan the transfer route, required equipment, medications, and emergency response.  ▸ Identify personnel involved.  ▸ Communicate the plan to the parents. |
|  | ICU Doctor & Nurse conduct pre-transfer briefing  ↓ | **2nd Communication** | ▸ Provide an update on the patient's status.  ▸ Announce imminent departure from ICU.  ▸ Confirm the assigned transport team is ready. |
|  | ICU Nurse calls to ensure elevator is ready  ↓ |  |  |
| **En Route** | ICU Doctor, Nurse, & Porter transport the patient  ↓ | **Monitoring** | The ICU nurse is responsible for monitoring the patient's vital signs, all lines/tubes, and equipment function during transport. |
| **Arrival at Target Dept.** | ICU Nurse & Ward Nurse settle the patient  ↓ | **Patient Placement** | ▸ Transfer the patient to the ward bed.  ▸ Connect monitors and other necessary equipment.  ▸ Manage all lines and tubes. |
|  | **Conduct Handover Timeout**  ↓ | **Pause** | ▸ Both the transport and receiving teams pause other activities to focus entirely on the handover. |
|  | ICU Doctor & Nurse give handover report  ↓ | **Handover** | ▸ **Patient Information:** Basic details, severity of illness.  ▸ **Transport Summary:** Events during transport.  ▸ **Clinical Plan:** Treatment plan, skin integrity, status of lines/tubes, medication schedule, nursing care plan. |
|  | Ward Nurse & Doctor ask questions  ↓ |  |  |
|  | Both parties confirm handover content  ↓ |  |  |
|  | **Transfer & Handover Complete**  ↓ | **End** |  |
|  | Document the handover | **Documentation** |  |

**Recommendation 4** Implement intrahospital transport of hospitalized children in accordance with standardized transport procedures. (1D)

**Recommendation 5** The standard intra-hospital transport procedure includes, but is not limited to, the following steps:

a) Nurses and physicians jointly assess the patient’s transport risks and potential benefits. After fully informing the parents, the decision to proceed with transport is made collaboratively between the healthcare team and the family.
b) Prior to transport, adequate coordination and communication should occur between the sending and receiving departments.
c) The transport route should be planned in advance.
d) A transport preparation checklist should be used to guide all preparatory activities for intra-hospital transport.
e) Upon arrival at the target department, personnel from both the sending and receiving units should perform the handover using standardized checklists.
f) Before the handover begins, a pause procedure should be implemented to allow all involved personnel to focus on the handover without distractions. Once the handover starts, interruptions should be avoided. Sufficient time should be provided for the receiving staff to ask questions. The handover is considered complete only after both sending and receiving personnel confirm the accuracy of the transferred information.
g) The patient’s condition and all medical interventions during transport must be clearly documented in the medical record. (1D)

**Recommendation 6** Body weight is a major factor influencing the ability of neonates to maintain normothermia after returning to the NICU. Waiting for surgery and undergoing diagnostic procedures are critical phases for hypothermia in NICU neonates. (2B)

**Recommendation 7** Effective thermal protection measures should be implemented during neonatal intrahospital transport. (1D)

**Tools and Methods for Implementing Recommendations:** Providing effective thermal protection to neonates requiring transport can reduce vital sign fluctuations during transport and improve clinical outcomes. Recommended warming measures include, but are not limited to, heated mattresses, warming packs, thermal clothing, and specially designed transport incubators.

**Recommendation 8** Specially designed intrahospital transport trolleys can improve the safety of neonatal transport. (2D)

**Recommendation 9** For children with diabetic ketoacidosis receiving continuous insulin infusion, blood glucose should be monitored within 30 minutes prior to intrahospital transport. (2C)

**Recommendation 10** Children receiving mechanical ventilation after cardiac surgery should be transported intrahospitally using a mobile ventilator. (2D)

**Recommendation 11** Intrahospital transport can be performed for children receiving extracorporeal membrane oxygenation (ECMO) support. (2D)

**Recommendation 12** High-quality cardiopulmonary resuscitation (CPR) during intrahospital transport is a prerequisite for ECMO rescue in children who experience cardiac arrest in non-ICU settings. (2D)
