# Supplementary material for: Effect of implementing an evidence-based clinical practice intra-hospital transport guideline for hospitalized children: a type III hybrid implementation-effectiveness study
Source: Implement Sci Commun. 2026 Jun 6;7:168. doi: 10.1186/s43058-026-00962-7 (PMC13508307; doi:10.1186/s43058-026-00962-7)
Supplement: Supplementary file 3 [file 43058_2026_962_MOESM3_ESM.docx]

**Electronic Supplement Material 3. ERIC Implementation Strategies and Corresponding Change Measures (n=24)**

| Theme | Strategy | Definition | Actor | Action | Target | Timing | Dose |
| --- | --- | --- | --- | --- | --- | --- | --- |
| Guiding Principle | Facilitation | The process of collaboratively solving problems and providing support within the context of recognized improvement needs and supportive interpersonal relationships | | | | 2024.07.01-2025.06.30 | Continuous |
| Guiding Principle | Adaptive Facilitation | Clarifying and maintaining essential elements of change while identifying methods to adapt the intervention to local needs | | | | 2024.07.01-2025.06.30 | Continuous |
| Developing Stakeholder Interrelationships (n=7) | Identify and Prepare Champions | Identify and prepare facilitators to support guideline implementation | Project Team | 1.Select personnel from clinical teams with leadership, communication, and change management skills; 2.Provide prioritized training for facilitators; 3.Facilitators promote, guide, and assist staff in implementing graded intrahospital transport during department meetings and daily work | Implementation Facilitators | 2024.08.26-2025.06.30 | Continuous |
|  | Identifying Early Adopters | Identify early adopters and understand their experiences | Project Team | 1.Identify departmental early adopters who are open to new technologies, processes, or innovations and willing to trial them first, by consulting unit leaders and reviewing behavioral records; 2.Conduct interviews to gather their experiences, insights, and recommendations regarding changes in intra-hospital patient transport. | Early Adopters | 2024.08.26-10.31 | Continuous |
|  | Involve Executive Boards | Secure leadership involvement in implementation | Nursing Department and Project Leads | Invite the Nursing Department, Quality Control Office, Medical Affairs Department, and unit directors to participate in the implementation. Quality control team members will routinely use quality assessment tools to review implementation data at all levels. | Leadership | 2024.08.12-2025.06.30 | Continuous |
|  | Inform Local Opinion Leaders | Inform and leverage opinion leaders to influence other participants | Project Team | 1.Identify positive opinion leaders or "educationally influential" staff (e.g., department heads, head nurses, clinical specialists, educators) with extensive social networks, professional expertise, and ability to interpret and promote innovations through consultations with leadership and peers; 2.Prioritize these opinion leaders for initial training; 3.Empower them to actively champion and model guideline-based intra-hospital transport practices within their units, demonstrating practical benefits and building colleague confidence in the change. | Local Opinion Leaders | 2024.08.26-2025.06.30 | Continuous |
|  | Model and Simulate Change | Conduct pre-implementation simulations to assess impact | Simulation Trainer | 1.Develop simulation-based training curricula, prepare clinical scenarios, and assemble required materials;2. Conduct live simulation exercises, observe healthcare staff performance, and provide structured feedback and guidance. | Clinical Staff | 2024.09.23-10.27 | Once a week |
|  | Capture and Share Local Knowledge | Collect and disseminate on-site success experiences | Implementers | 1.Conduct participatory observation to document key implementation steps, challenges encountered, and solutions developed, while collaborating with implementers to gain deep insights into their experiences and methods. 2.Implementers share specific patient transfer cases during departmental meetings to facilitate peer learning. | Local Knowledge Sharing | 2024.09.01-10.31 | Once a week |
|  | Conduct Local Consensus Discussions | Facilitate stakeholder discussions on the clinical problem's importance and the change's appropriateness | Clinical Experts | Organize clinical experts to discuss the significance of current patient transport workflow issues and adapt the change protocol based on literature review and clinical context to achieve stakeholder alignment. | Clinical Problem & Change Adaptation | 2024.07.01-10.31 | At least twice |
| Use Evaluative and Iterative Strategies (n=6) | Develop and Implement Tools for Quality Monitoring | Develop and implement quality assessment standards | Project Team & Nursing Quality Control Team | 1.Design and develop quality monitoring standards and tools, including intra-hospital transport audit forms and adverse event reporting forms; 2.Integrate process and outcome measures of patient transport into the nursing quality control management system. | Transport Quality Metrics | 2024.08.12-2025.06.30 | Continuous |
|  | Assess for Readiness and Identify Barriers and Facilitators | Evaluate organizational dimensions to determine implementation readiness, barriers, and facilitators | Project Team | 1.Conduct interviews and/or field observations to assess unit resource availability, staff knowledge/skill levels, training needs, and inter-departmental collaboration; 2.Analyze qualitative data to identify potential barriers and facilitators to guideline implementation. | Barriers & Facilitators to Transport Implementation | 2024.07.08-07.14；2024.10.01-10.06 | Carry out twice |
|  | Conducting Local Needs Assessment | Collect data relevant to change requirements | Project Team | 1.Analyze historical unit quality reports and patient safety incident reports to identify risks and deficiencies in current transport processes; 2.Perform direct observations of current transport workflows to detect potential issues and improvement needs; 3.Administer surveys and interviews to gather staff feedback on existing transport procedures, along with their awareness and acceptance of guidelines. | Local Change Requirements | 2024.07.01-07.31 | Continuous |
|  | Develop a Formal Implementation Blueprint | Develop a structured implementation plan | Project Team & Leadership | 1.Establish a detailed timeline with specific phase objectives, tasks, and outcome metrics. 2.Conduct weekly progress reviews within the research team against the blueprint. | Implementation Blueprint | 2024.08.12-10.31 | Once a week |
|  | Audit and Feedback | Systematically collect audit data and provide feedback to leadership and providers | Project Team & Quality Control Team | 1.The Project Team conducts ongoing data collection throughout the study period; 2.Quality control team members perform regular audits at all levels during and post-study, delivering findings to clinical staff and leadership. Nursing and Medical Affairs departments summarize, discuss, and refine hospital-wide patient transport quality control measures. | Clinical Audit Data | 2024.09.01-2025.06.30 | Weekly review and monthly meetings |
|  | Purposefully Reexamine the Implementation | Monitor progress and adapt clinical practices and implementation strategies | Project Team & Quality Control Team | Periodically review the change initiative's progress and adjust implementation strategies based on audit findings. | Implementation Strategies | 2024.09.01-2025.06.30 | Once every three months |
| Support Clinicians (n=1) | Facilitate relay of clinical data to providers | Communicate key performance metrics to clinical staff through established channels | Unit Leadership | 1.Present audit findings during morning briefings; 2.Share analyzed results through unit-managed communication platforms; 3.Display performance data on staff information boards. | Clinical Audit Metrics | 2024.09.01-2025.06.30 | Continuous |
| Adapt and Tailor to Context (n=1) | Tailor Strategies | Develop implementation strategies to address identified barriers and leverage facilitators | Project Team | Customize implementation strategies to resolve barriers and capitalize on facilitators identified through preliminary data collection. | Implementation Strategies | 2024.08.01-08.18； | Continuous |
| Train and Educate Stakeholders (n=6) | Make Training Dynamic | Employ multiple interactive information-delivery methods for training | Project Team & Clinical Experts | Implement diverse interactive training formats including: distributing educational materials, conducting online and in-person theoretical sessions, and organizing live simulation exercises. | Training | 2024.08.26-2024.10.31 | Continuous |
|  | Developing Educational Materials | Create and design manuals, toolkits, and supplementary resources | Transport Guideline Development Experts, Critical Care Specialists, & Project Team | 1.Produce print and digital educational manuals containing: guideline background, recommendation interpretations, local consensus summaries, and instructions for all tools/checklists; 2.Update existing intra-hospital transport protocols according to guideline specifications; 3.Develop rapid-reference toolkits including: transport algorithms, risk assessment tools, equipment checklists, and portable SBAR handoff tools. | Educational Materials | 2024.08.19-08.31 | Continuous |
|  | Distributing Educational Materials | Disseminate educational resources through multiple channels | Project Team & Unit Leadership | 1.Coordinate with unit leaders to distribute materials to clinical staff prior to training sessions; 2.Promptly circulate updated materials with full notification, conducting unit-level training when necessary; 3. Consolidate digital resources on an accessible platform for on-demand staff access and learning. | Educational Materials | 2024.08.26-09.30 | Continuous |
|  | Conducting Educational Meetings | Deliver targeted training for distinct stakeholder groups | Project Team & Education Specialists | 1. Convene an expert training team including critical care specialists, implementation experts, and guideline developers; 2. Employ multiple formats: in-person workshops, web-based seminars, and topical lectures; 3.Train unit leadership on core guideline elements, implementation importance, resource allocation, and performance evaluation; 3.Educate clinical staff on guideline content, operational procedures, and equipment utilization. | Training Sessions | 2024.08.26-09.30 | Once a week |
|  | Conduct Educational Outreach Visits | Train providers in their practice environment | Simulation Specialists & Trained Senior Clinicians | 1. Engage dedicated simulation specialists and trained senior clinicians to design and deliver on-site training programs; 2. Trainers demonstrate transport procedures in clinical units, provide real-time implementation guidance with corrective feedback, and address practice-related questions. | On-Site Coaching | 2024.09.01-10.31 | Continuous |
|  | Shadow Other Experts | Key personnel observe experienced providers implementing change | Demonstrators | 1. Select demonstrators (e.g., facilitators, early adopters, opinion leaders) who play crucial roles in the change effort and possess extensive transport experience; 2. Form observation teams comprising new staff and providers with lower protocol adherence, flexibly organizing them to observe demonstrators' intra-hospital transport activities during clinical downtime. | Observers | 2024.09.23-10.31 | Continuous |
| Changing Infrastructure (n=1) | Change Physical Structure and Equipment | Assess existing configurations and adapt infrastructure to meet needs | Hospital Administration & Engineering/ Maintenance Departments | 1. Conduct detailed audits of existing transport equipment to assess compliance with change requirements, updating or augmenting resources based on findings; 2. Evaluate unit physical layouts to optimize transport route accessibility and safety; 3. Implement clear wayfinding signage and path mapping throughout the facility. | Spatial Configuration | 2024.08.01-2025.06.30 | Continuous |
